# Supplementary material for: Complexome profiling of the Chlamydomonas psb28 mutant reveals TEF5 as an early PSII assembly factor
Source: Plant Cell. 2025 Mar 17;37(6):koaf055. doi: 10.1093/plcell/koaf055 (PMC12164590; doi:10.1093/plcell/koaf055)
Supplement: koaf055_Supplementary_Data [file koaf055_supplementary_data.zip › psb28_interactive.zip!/plots/intensity/qualityPlots/peptides/_Cre13.g576760.t1.1.html]

### Cre13.g576760.t1.1

Trivial name:   
SeqCoverage [median]: 9.70  
Mass: 38.84 kDa  
  
 

| Sequence | MissedCleavage | Mass [Da] | wtIntensity | muIntensity | Score |
| --- | --- | --- | --- | --- | --- |
| GLGDVVGMK | 0 | 874.46 | 4013.10 | 15492.22 | 104.37 |
| GLGDVVGMKGPAAEINNGR | 1 | 1853.94 | 0.00 | 728.09 | 70.78 |
| GPAAEINNGR | 0 | 997.49 | 0.00 | 2514.55 | 147.36 |
| LESPIVLPSGGDLKPK | 1 | 1648.94 | 5082.05 | 22341.75 | 122.93 |
| RLESPIVLPSGGDLKPK | 2 | 1805.04 | 3204.21 | 33891.51 | 104.88 |
|  |  |  | 12299.4 | 74968.1 |  |
